# Measuring body condition of lizards: a comparison between non-invasive dual-energy X-ray absorptiometry, chemical fat extraction and calculated indices

**DOI:** 10.1186/s12983-020-00382-w

**Published:** 2021-01-05

**Authors:** Guy Sion, Maggie J. Watson, Amos Bouskila

**Affiliations:** 1 Department of Life Sciences, Ben-Gurion University of the Negev, P.O. Box 653, 8410501 Beer Sheva, Israel; 2 Present address: School of Zoology, Faculty of Life Sciences, Tel Aviv University, 69978 Tel Aviv, Israel; 3 Present address: Institute for Land Water and Society, Charles Sturt University, P.O. Box 789, Elizabeth Mitchell Drive, Albury, NSW 2642 Australia; 4 Mitrani Department of Desert Ecology, Blaustein Institutes for Desert Research, Ben Gurion University of the Negev, 8499000 Midreshet Ben-Gurion, Israel

## Abstract

**Background:**

Condition indices (CIs) are used in ecological studies as a way of measuring an individual animal’s health and fitness. Noninvasive CIs are estimations of a relative score of fat content or rely on a ratio of body mass compared to some measure of size, usually a linear dimension such as tarsus or snout-vent length. CIs are generally validated invasively by lethal fat extraction as in a seasonal sample of individuals in a population. Many alternatives to lethal fat extraction are costly or time consuming. As an alternative, dual-energy X-ray absorptiometry (DXA) allows for non-destructive analysis of body composition and enables multiple measurements during an animal’s life time. DXA has never been used for ecological studies in a small, free-ranging lizard before, therefore we calibrated this method against a chemical extraction of fat from a sample of 6 geckos (Israeli fan toed gecko *Ptyodactylus guttatus)* ranging in body mass between 4.2–11.5 g. We then  used this calibrated  DXA measurements to determine the best linear measurement calculated CI for this species.

**Results:**

We found that fat mass measured with DXA was significantly correlated with the mass of chemically extracted fat for specimens more than 4.8 g (*N* = 5, *R*^2^ = 0.995, *P* < 0.001)*.* Fat percentage regressed with body mass significantly predicted the DXA fat percentage (*N = 29, R*^*2*^_*adj.*_ *= 0.862, p < 0.001*). Live wet mass was significantly correlated with predicted fat mass (*N* = 30, *R*^2^ = 0.984, *P* < 0.001) for specimens more than 4.8 g. Among the five calculated non-invasive CIs that we tested, the best was mass/SVL.

**Conclusions:**

We recommend that in situations where DXA cannot be used, that the most accurate of the body condition estimators for  this species is mass/SVL (snout-vent length) for both sexes.

## Background

Body condition is a term used by ecologists to rank the ‘quality’ of an individual animal, usually in relation to the amount of energy reserves (usually fat stores) an animal has available [17]. Many animal ecology studies rely on destructive or estimation methods to determine the body condition of different individuals in a population [37]. Both destructive (body composition) and non-destructive (body mass and linear measures of body size) are used to estimate or determine condition indices (CI) of an individual. Body condition is assumed to influence an animal’s health and fitness and may affect many aspects in an organism’s life such as social status (dark-bellied brent geese *Branta bernicla bernicla*, [23]), reproductive success (crimson finch *Neochmia phaeton*, [19]), foraging strategy (white-tailed deer *Odocoileus virginianus*, [38]; meerkat *Suricata suricatta,* [39]), survival through stressed periods (golden-mantled ground squirrel *Callospermophilus lateralis*, [42]), disease status (green sea turtle *Chelonia mydas*, [26]) and dispersal (viviparous lizard *Zootoca vivipara*, [18]). It is highly desirable to understand body condition, both temporally and ontogenetically, in order to provide supporting evidence and mechanistic linkages for population studies [37]. In population studies of species of conservation importance, new and improved non-destructive methods for body condition indices are increasingly important [22, 34].

Body fat, due to its high energy content, is the best measure of body condition of an animal [10]. Body fat reserves directly influence fitness and are highly dependent on season, reproductive status and periods of fasting (e.g., [2, 35]). The most accurate measure of body fat is the direct approach wherein several individual animals are euthanized and have their fat extracted chemically from the carcasses (e.g., [24, 49]). This destructive approach is, however, complicated, time consuming and does not allow for comparisons of body condition within and between seasons on the same individuals. Non-destructive techniques include various body condition estimators (ratio of body mass to a linear dimension of body size, or the residuals of the regression between body mass and size [9, 28];), isotope dilution [29], bioelectrical impedance analysis [13], total body electrical conductivity (TOBEC, [1, 27]), lipid-soluble gas absorption [12], quantitative magnetic resonance (QMR) [25, 41], and dual-energy X-ray absorptiometry (DXA) [20].

Bioelectrical Impedance Analysis (BIA) appears to be a better predictor of body fat than body condition estimates calculated from mass and SVL [43]. However, the repeatability and accuracy are not sufficient to monitor small changes in Lean Body Mass (LBM) and lipid stores [28]. Among the alternative techniques, DXA, holds the most promise as an easy and accurate measure, especially for smaller animals. DXA scans the body with two X-ray beams of different energy levels and uses the attenuation of the energy of those two X-ray beams to determine the tissue signature and to quantify total body mass, lean mass and fat mass of the organism [16].

DXA has been used to assess nutritional status in captive rhesus monkeys *Macaca mulatta* (compared with stable isotope dilution, no validation, [3]); measure fat mass in small migratory birds (validated with freeze-dried carcasses, [16]), identify metabolic bone disease and bone mineral density in captive green iguanas *Iguana iguana* (no validation, [50]), determine the body composition of diamondback water snakes *Nerodia rhombifer* (validated with euthanized individuals, [28]) and channel catfish *Ictalurus punctatus* (validated with euthanized individuals, [14]). In reptiles, body condition is generally estimated by morphometric measurements [11, 37] which are rarely tested against other CIs or validated (but see [5, 28]). In some studies, morphometric measurement based CIs were correlated with locomotor performance, but  proved to be poor predictors of maximum speed and exertion (Vervust et al. 2008 [40]). The use of DXA has not been tested for its applicability for studies of small lizards, thus our primary objective is to assess the use of DXA as a practical method for determining body fat composition, and therefore use as a CI, in a small lizard. Our specific goals are (1) to evaluate the accuracy of DXA in predicting body fat composition of a small lizard by comparing DXA to chemically extracted fat, (2) to compare the accuracy of this method for males, females and juveniles, and (3) to quantify the relationship between five common estimators of body condition (based on mass and/or length relationships) with body fat estimated from the validated DXA analysis.

## Methods

### Study site and organism

The study was conducted at Midreshet Ben-Gurion in the northern Negev desert, Israel (30°51′8.27″N 34°47′0.24″E) from summer 2003 until autumn 2004. The study site was a complex of guest rooms surrounded by a two-meter high wall over an area 13x150m. A dense population of the Israeli fan-toed gecko *Ptyodactylus guttatus* inhabited the premises [30–32]. The Israeli fan-toed gecko is a medium-sized, insectivorous, rupicolous, scansorial lizard [6, 45] Zlotkin et al. 2003 [50], Sion et al. 2020 [33] in the family Phyllodactylidae [7] common in mesic and arid parts of the Middle East (Israel, Egypt, Saudi Arabia, Oman, Palestine, Jordan, and Syria). It often inhabits cliffs or masonry walls where it can easily be observed from a distance ([48, 47], 2016 [6, 15, 30–32, 46]).

Fifty-five geckos were hand captured, measured (morphometrics) and scanned (DXA) and released at the site of capture. Of these, 30 gecko’s scan data were included in the comparison, since their body mass was above the lowest possible accurate reading with minimal body mass (> 4.8 g) as indicated in the results (Table 1). The snout vent length (SVL) of these 30 geckos was 60.6–91.7 mm and their body mass 4.99–22.5 g. We used these 30 geckos to compare the real wet mass (as measured by a scale) and the wet mass measure by DXA (see below). From each captured gecko, we recorded the mass using *Ohaus* digital scale to 0.1 g precision, snout-vent length (SVL), using digital calipers, and the width at the base of the tail. Six additional individuals were captured and euthanized for the calibration necessary for this study (two males, three females and one too small to be sexed without a probe) The smallest gecko (55.6 mm) with body mass 4.2 g was excluded to improve accuracy from 55 to 8.5% error. The snout vent length (SVL) of these geckos was 61.5–91.7 mm and their body mass 4.8–11.5 g. We killed only six geckos in order to minimize destructive sampling as much as possible.

**Table 1:** Sex, snout-vent length (SVL), live wet body mass, DXA mass reading and fat mass chemical extraction of six Israeli fan-toed geckos *Ptyodactylus guttatus* used to validate the application of dual-energy X-ray absorptiometry (DXA) to non-invasively calculate body fat indices in small lizards

Sex	SVL (mm)	Live Wet Mass (g)	DXA Mass Reading (g)	Fat Extraction (g)	DXA Fat Reading (g)
Male	61.51	4.8	5.7	0.26	1.5
Male	87.45	11	12	0.37	1.65
Female	80.74	10.9	11.9	1.60	2.4
Female	65.97	11.5	13	2.09	2.8
Female	67.14	6.5	7.6	0.93	2
Unknown^a^	55.64	4.2	5.2	0.43	0.95

^a^ Indicates the lizard that was removed from the validation experiment (see text for details)

### Dual-energy X-ray absorptiometry (DXA) measurements

Geckos were anesthetized using cotton balls wet with Isofluran which was inserted with the gecko in a sealed jar for a minute or less until the gecko stopped moving. To obtain the DXA measurements, each gecko was scanned twice using a Lunar PIXImus® 2 densitometer (software version 1.46, originally manufactured for scanning rodents, e.g., [21]). Each scan takes approximately 5 min, and requires that the animal remains immobile for the duration of the scan [28]. For each lizard we averaged the values we measured in the two scans. Scans were analyzed using the manufacturer’s software that provided a measurement of fat mass, fat percentage and lean mass. These 30 scan results were also used to compare the measured (calibrated) body fat percentage with the different indices for body condition. Immediately after DXA analysis, the six individuals chosen for chemical analysis were killed with an overdose of Isofluran and frozen at − 20 °C until analyzed for carcass composition.

### Chemical analysis

The DXA machine protocol was designed for 10–50 g. laboratory mice [21], but it has been successfully used for other species such as large mammals [35], small mammals [36], and small birds [16] to measure fat content and body composition. We decided to examine the performance of the DXA machine for smaller size animals to find the lower limit of the machine’s detection abilities. The six euthanized geckos were kept frozen and then were freeze-dried until constant mass was achieved (~ 2 days). The dried body was then ground and extracted with petroleum ether in a Soxhlet apparatus to determine fat mass using the methods of Dobush et al. [4] with guidance from Noga Kronfeld-Schor (following Gutman et al 2006 [8]). In short, a small sample was taken from each ground carcass, inserted into a (tared) empty tea bag and weighed on analytical scales (empty bags supplied by Lipton™). Each sample was weighed ±0.0001 g. on an analytical balance (Ohaus), in the tea bag before fat was washed out by the organic solvent petroleum ether. The fat was washed out during repetitive washes, using a set of glass tubes that enabled the solvent that washed fat into a glass jar to evaporate by heat and then reliquidize apart from the samples. The solvent was recycled for repetitive washes for 2 days.

### Data analysis

We used backward selection mode of a step-wise regression to test the relation between DXA values of body mass and the values determined from the chemical analysis of body composition. Single and multivariate regression models were then constructed to predict the fat mass from DXA readings. We report the results of our statistical analysis in terms of their *R*^2^ and *P* values. For statistical analysis, we used SPSS (version 20).

## Results

### DXA validation

**Fig. 1:**
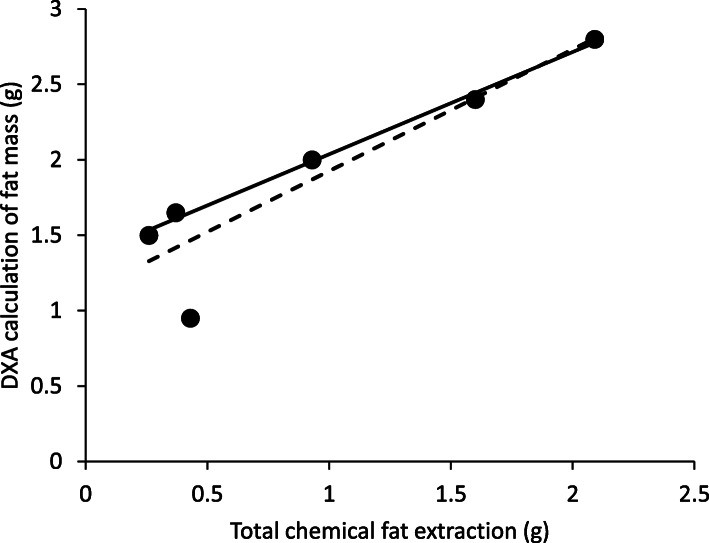
Correlation between actual total fat as determined by chemical extraction and calculation of fat mass as determined by DXA (dashed line including the smallest lizard: Linear regression: *R*^*2*^ = 0.834; *y* = 0.808*x* + 1.119); solid line excluding the smallest lizard (Linear regression: *R*^2^ = 0.995; *y* = 0.679*x* + 1.357)

Six geckos were used for the validation portion of this study (Table 1). The values of fat mass as determined by DXA (raw data) were highly correlated to the chemically extracted fat mass values (Linear regression: *N* = 6, *R*^2^ = 0.834, *P* = 0.011; Fig. 1). Removing the smallest (in both SVL and mass), unsexed gecko improved the error from 54 to 8.5% (Linear regression: *N* = 5, *R*^2^ = 0.995, *P* < 0.001; Fig. 1). We found a linear correlation (*R*^*2*^ = 0.995) and a lack of significant difference between fat content by chemical extraction and by DXA estimation (Paired *t-test*: *t*_*4*_ = − 0.03; *P* = 0.998).

### Accuracy of DXA for calculating mass

**Fig. 2:**
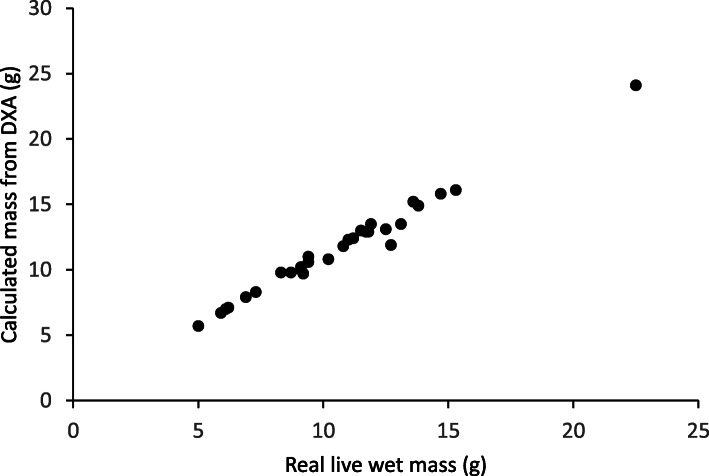
The values of body mass taken from the DXA machine as raw data (as is) vs. body mass values as measured by scales. Linear regression: *N* = 30, *R*^2^ = 0.984; *y* = 1.019*x* + 0.804

To determine the accuracy of the DXA in calculating mass compared to actual weighed mass, we compared all lizards above 4.8 g. The DXA body fat readings were highly correlated to live wet mass (linear regression: *N* = 30, *R*^2^ = 0.984, *P* < 0.001; Fig. 2) and chemically extracted fat mass: *N* = 5, *R*^2^ = 0.995, *P* < 0.001.

### Models of fat percentage and mass

**Fig. 3:**
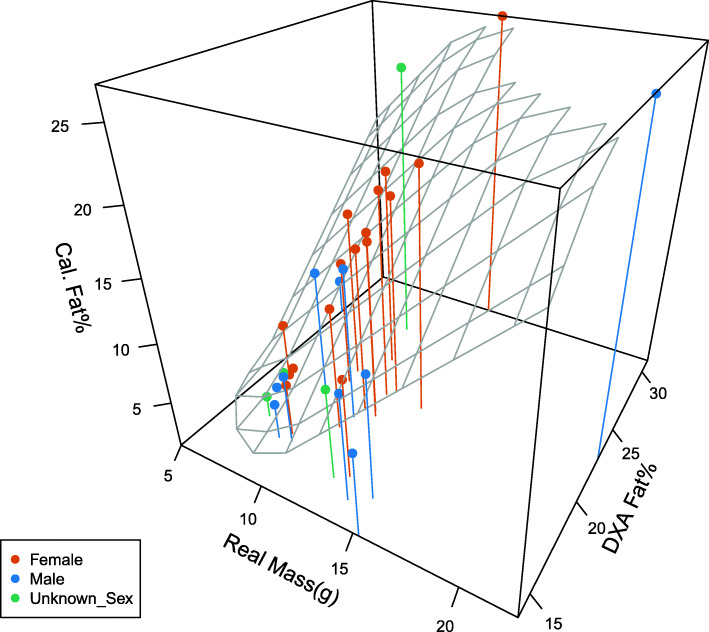
A multivariate model of DXA calculated fat percentage (DXAFat %), calculated fat percentage (Cal. Fat %) and actual measured live wet mass (Real Mass (g)) for male (*N =* 10), female (*N =* 16) and unsexed (*N* = 4) Israeli fan-toed geckos. *N* = 30, *R*^*2*^_*adj*._ = 0.836, *P* < 0.001, y = − 30.412 ± 3.669 + 1.523 ± 0.157(x_Fat%_) + 1.289 ± 0.151(x_Mass_). When removing the outlier male (*N =* 29), the model improves to *R*^2^_adj._ = 0.862, *P* < 0.001, y = − 36.341 ± 3.644 + 1.404 ± 0.134(x_Fat%_) + 1.903 ± 0.179(x_Mass_)

A simple linear regression of calculated (using the formula from the validation data) vs. DXA machine’s predicted fat percentage was significant (*P =* 0.045) but the percentage of explained variation was low (*R*^2^ = 0.136). We regressed a multivariate regression model, constructed with mass and DXA machine fat percentage (not calibrated) as variants to predict the correct fat percentage: *N* = 30, *R*^*2*^ = 0.847, *R*^*2*^_*adj*._ = 0.836, *P* < 0.001; y = − 30.412 ± 3.669 + 1.523 ± 0.157(x_Fat%_) + 1.289 ± 0.151(x_Mass_) (Fig. 3). We excluded one outlier, a male that was more than 3SD from the mean (22.5 g, 91.65 mm SVL with a calculated fat percentage of 60.8% while the remaining 29 ranged from 0.7–34.4%), resulting in an improved model: *N =* 29, *R*^*2*^ = 0.872, *R*^*2*^_*adj*._ = 0.862, *P* < 0.001; y = − 36.341 ± 3.644 + 1.404 ± 0.134(x_Fat%_) + 1.903 ± 0.179(x_Mass_).

Average total body wet mass was 11.1 ± 7.0% body fat (*N* = 29), and did not significantly depart from a normal distribution (Kolmogorov-Smirnov test; *Z* = 0.431, *D* = 0.080*, p =* 0.992). Females tended to have a higher percentage of their body mass as fat (13.21 ± 7.17, *N* = 16) compared to males (7.94 ± 4.19); however, this difference was not significant (*t-test*, *P* = 0.056).

### Comparisons of DXA to calculated CI

We applied five different morphological condition indices that are commonly used on reptiles to the 30 geckos in the dataset in order to determine their accuracy. The indices tested were 1) width of tail base; 2) ordinary least squares (OLS) linear regression of body mass (g) against SVL (mm); 3) mass/SVL; 4) mass/SVL^2^; 5) mass/SVL^3^.

All body indices were highly correlated with each other (*P* < 0.05). The correlations between calibrated body fat percentage and body condition indices was largest for the mass/SVL index. The correlation coefficient was highest for the Mass/SVL index > mass/SVL^2^ > width of tail base > Mass/SVL^3^ > OLS (Table 2). When correlating separately by sex, the width of tail base was best in males (*r*_tail base width_ = 0.722, *P* < 0.043) and not significant for females.

**Table 2:** Pearson correlation coefficient test results for the relationship between DXA calculated body fat percentage (using the *N* = 29, *R*^2^_adj._ = 0.862 model) and body condition indices for male and female Israeli fan-toed geckos *Ptyodactylus guttatus*

		Calculated Fat %	Width of Tail Base	OLS	Mass/SVL	Mass/SVL^2^	Mass/SVL^3^
Calculated Fat Percentage	Pearson Correlation	1	.398*	.346	.592**	.499**	.290
	Sig. (2-tailed)		.044	.066	.001	.006	.128
	N	29	26	29	29	29	29
Width of Tail Base	Pearson Correlation	.398*	1	.047	.531**	.249	−.105
	Sig. (2-tailed)	.044		.819	.005	.220	.609
	N	26	26	26	26	26	26
OLS	Pearson Correlation	.346	.047	1	.744**	.942**	.959**
	Sig. (2-tailed)	.066	.819		.000	.000	.000
	N	29	26	29	29	29	29
Mass/SVL	Pearson Correlation	.592**	.531**	.744**	1	.904**	.624**
	Sig. (2-tailed)	.001	.005	.000		.000	.000
	N	29	26	29	29	29	29
Mass/SVL^2^	Pearson Correlation	.499**	.249	.942**	.904**	1	.897**
	Sig. (2-tailed)	.006	.220	.000	.000		.000
	N	29	26	29	29	29	29
Mass/SVL^3^	Pearson Correlation	.290	−.105	.959**	.624**	.897**	1
	Sig. (2-tailed)	.128	.609	.000	.000	.000	
	N	29	26	29	29	29	29

** Correlation is significant at the 0.01 level (2-tailed)

* Correlation is significant at the 0.05 level (2-tailed)

## Discussion

Non-invasive and non-destructive techniques to determine the condition of individual animals are preferential to destructive methods requiring euthanasia, especially for rare and endangered species or in cases of strict laws concerning wildlife protection or animals’ rights laws. However, these sorts of longitudinal measures allow for greater understanding of life-history traits and behavior and are therefore vital for scientific study. A variety of non-invasive techniques have been trialed, at varying levels of success, to measure fat stores in reptiles, but none have been tested and verified on small free-ranging lizards. This study aimed to determine whether dual-energy X-ray absorptiometry (DXA), a non-destructive method that accurately measures fat mass in humans and other mammals, could be used to determine the fat mass in the small, common Israeli fan-toed gecko. We found that the lowest value for body mass calculation by the DXA machine and that was still accurate was 4.8 g. Thus, when we exclude the smallest gecko (4.2 g), we found that DXA and chemical extraction of fat mass were significantly correlated. It should be stressed that the validation sample size was small and there were not enough individuals to explore sex differences, so further exploration of the technique is warranted. Regression equations using DXA values were able to predict the fat mass and total gravimetric body mass accurately with an average error of 8.55 and 3.5% respectively. Using the predicted DXA values, we were able to test common body condition indices against both actual and predicted DXA values and show that mass/SVL was the most accurate method for estimating the condition index of these small lizards. Measuring the tail base width was not correlated with females body percentage; however for males, it was more accurate than mass/SVL. Females and males may differ in their patterns of fats storage, which might explain why the tail base width was not correlated with fat percentage in females, while it was the best indicator for fat percentage in males.

### Non-destructive methods for CI

Previous work on reptile body condition indices and non-destructive sampling have been notoriously inaccurate. Secor and Nagy [28] found that two indices for body condition (mass/SVL and mass/SVL^2^) predict the fat mass of diamondback water snakes (*Nerodia rhombifer*) with significantly different values from the DXA and chemical analysis results (mean error ranging from 20 to 41%). Weatherhead & Brown [44] found another index method (based on log of mass residuals) to be relatively unreliable in predicting fat mass of northern water snakes (*N. sipedon*). Other methods for the estimation of fat percentage (e.g., Bioelectrical Impedance Analysis) have been fairly successful in predicting body condition, but again they lack accuracy ([28]). Total body electrical conductivity (TOBEC) is an additional technique and is relatively simple, however, it is sensitive to body size, shape, temperature and hydration state, as well as distribution of lean tissue. For the eastern fence lizard *Sceloporus undulatus*, TOBEC estimates of fat free dry mass and fat mass were able to predict the actual amounts present with an average error of 5.8 and 30.3%, respectively. Of all the non-invasive methods used to quantify body composition, the most accurate are QMR ([41]; using the brown anole *Anolis sagrei* found a predicted fat error of 4.5%*,* but note that [25] found the method had large predicted fat errors (80%) in northern watersnakes *Nerodia sipedon sipedon* and eastern massasaugas *Sistrurus catenatus catenatus*) and DXA (this study). The mean error percentage for fat mass we calculated for using DXA was 8.55% when the animal body mass was more than 4.8 g. This percentage designated the difference between the fat mass predicted by the model and the fat mass that was extracted chemically. The mean error of the wet body mass was even lower, at 3.5%. Thus, the body index method we used—measurement of body fat% by DXA— was proved to be most reliable and highly correlated to chemically extracted body fat.

### Practical use of DXA and CI

We found that DXA was a reliable rapid and accurate means to predict fat mass and body mass in live small lizards. It enables a researcher to accurately track seasonal and ontogenetic changes in an individual and to correlate these changes to fitness traits. The DXA machine and software were easy to use, and such machines are widely available due to DXA use in human medicine. The cost for such a machine solely for use on wildlife would be prohibitive (in 2001, this machine cost USD$110,000), but as a secondary use for machines already purchased for medical or veterinary laboratories, DXA becomes a cost-effective option. While cost is always a drawback for DXA, we suggest its primary benefit is to select the most accurate CI of the non-invasive methods. A validation study is required to generate the predictive models necessary to know which CI is the most accurate; the sacrifice of study animals becomes then a serious liability for studies on rare species. Another disadvantage is the necessity that subjects remain absolutely still, which requires anesthesia. In a pilot trial, we attempted to place the lizards in the freezer prior to the trial to prevent them from moving during the scanning phase, but this proved unreliable and had to be substituted with an anesthesia which can prove fatal in overdose.

As a final note on practicalities, when comparing five common traditional CI’s, we found that mass/SVL was the index with the highest correlation coefficient to the DXA estimates. We conclude that when DXA is not available, mass/SVL is the most reliable alternative index for this species and should be used unless actual fat mass is required. In situations where more accuracy is needed, DXA is a suitable method for small lizards. An interesting question raised here is the insignificant correlation between females tail base width and fat percentage, while in males it was the best predictor (*r* = 0.961). This result concerning tail width should be explored in other species of lizards in order to understand if this specific measurement is a general basic physiological difference between the sexes.

## Data Availability

Data are freely available from the paper and will be provided in a supplementary file.

## Abbreviations

CI: Condition index
DXA: Dual-energy X-ray absorptiometry
SVL: Snout-vent length
TOBEC: Total body electrical conductivity
